# Long Noncoding RNAs and mRNA Regulation in Peripheral Blood Mononuclear Cells of Patients with Chronic Obstructive Pulmonary Disease

**DOI:** 10.1155/2018/7501851

**Published:** 2018-03-13

**Authors:** Xiaoyan Qu, Xiaomin Dang, Weijia Wang, Ying Li, Dan Xu, Dong Shang, Ying Chang

**Affiliations:** ^1^Center for Translational Medicine, Frontier Institute of Science and Technology, and The Key Laboratory of Biomedical Information Engineering of Ministry of Education, School of Life Science and Technology, Xi'an Jiaotong University, Xi'an, China; ^2^Department of Respiration, The First Affiliated Hospital, Xi'an Jiaotong University, Xi'an, China

## Abstract

**Background:**

Inflammation plays a pivotal role in the pathogenesis of chronic obstructive pulmonary disease (COPD). We evaluated the lncRNA and mRNA expression profile of peripheral blood mononuclear cells (PBMCs) from healthy nonsmokers, smokers without airflow limitation, and COPD patients.

**Methods:**

lncRNA and mRNA profiling of PBMCs from 17 smokers and 14 COPD subjects was detected by high-throughput microarray. The expression of dysregulated lncRNAs was validated by qPCR. The lncRNA targets in dysregulated mRNAs were predicted and the GO enrichment was analyzed. The regulatory role of lncRNA *ENST00000502883.1* on *CXCL16* expression and consequently the effect on PBMC recruitment were investigated by siRNA knockdown and chemotaxis analysis.

**Results:**

We identified 158 differentially expressed lncRNAs in PBMCs from COPD subjects compared with smokers. The dysregulated expression of 5 selected lncRNAs *NR_026891.1 (FLJ10038)*, *ENST00000502883.1 (RP11-499E18.1)*, *HIT000648516*, *XR_429541.1*, and *ENST00000597550.1 (CTD-2245F17.3)*, was validated. The GO enrichment showed that leukocyte migration, immune response, and apoptosis are the main enriched processes that previously reported to be involved in the pathogenesis of COPD. The regulatory role of *ENST00000502883.1* on *CXCL16* expression and consequently the effect on PBMC recruitment was confirmed.

**Conclusion:**

This study may provide clues for further studies targeting lncRNAs to control inflammation in COPD.

## 1. Introduction

Chronic obstructive pulmonary disease (COPD) is characterized by progressive airflow obstruction that is only partly reversible, inflammation in the airways, and other systemic effects [1]. So far there is no satisfactory therapy to treat individuals once the disease is established. The mechanism of the disease generally involves aberrant, chronic inflammation coupled with the loss of lung structural cells due to heightened apoptosis.

Long noncoding RNAs (lncRNAs) are mRNA-like transcripts longer than 200 nucleotides without protein-coding functions [2]. According to the genomic proximity to protein-coding genes, lncRNA was generally classified into five types: (1) sense, (2) antisense, (3) bidirectional, (4) intronic, and (5) intergenic [3]. lncRNAs have been shown to play important roles in diverse biological and pathological processes and are dysregulated in various human diseases [4]. However, few studies concerning the dysregulation and regulatory role of lncRNA in lung diseases have been reported. Several hundred lncRNAs were identified in the developing mouse lung by sequencing polyadenylated RNAs in embryonic and adult lung tissue. *NANCI* and *LL34* were found to regulate expression of hundreds of genes in mouse airway epithelial cell culture, but their role in lung diseases remains unclear [5]. A recent study showed that lncRNAs including *RP11-46A10.4*, *LINC00883*, *BCYRN1*, and *LINC00882* act as miRNA “sponges” to regulate the growth of airway smooth muscle cells [6]. Bi et al. [7] analyzed lncRNA expression in the lung tissue of nonsmokers, smokers without COPD, and smokers with COPD; *RNA44121|UCSC-2000-3182* and *RNA43510|UCSC-1260-3754* were found the most over- and underregulated lncRNA, respectively.

Inflammation plays a pivotal role in the pathogenesis of COPD, where CD8^+^ T lymphocytes, neutrophils, and macrophages are the main type of immune cells of local inflammatory milieu of COPD [8]. Different immunoregulatory properties of T cells and monocytes have been demonstrated in COPD patients [9]. In the previous study, we have analyzed the expression profile of miRNAs as well as regulation network of dysregulated miRNAs and mRNAs in PBMCs of COPD patients [10]. Several studies on lncRNA expression of peripheral blood mononuclear cells (PBMC) in other diseases have been reported [11, 12]. However, the lncRNA expression profile of PBMCs in COPD patients remains undone.

In this study, we sought to determine if lncRNAs were differentially expressed in PBMC of patients with COPD and if lncRNA expression may be linked to dysregulated mRNA expression relevant to the pathogenesis of COPD. We analyzed lncRNA and mRNA expression profiles in PBMC from COPD patients versus nonsmokers and smokers without airflow limitation. The lncRNA targets in dysregulated mRNAs were predicted.

## 2. Methods

### 2.1. Subjects

Peripheral venous blood was taken in heparin-coated tubes from 20 healthy nonsmokers, 17 smokers without airflow limitation, and 14 COPD patients at the Department of Respiration, The First Affiliated Hospital, Xi'an Jiaotong University, Xi'an, China. The COPD patients were eligible for this study if they met the following criteria: age ≥ 50 and ≤76 years; smoking history (≥20 pack years); postbronchodilator FEV_1_ ≥ 25% of predicted value and postbronchodilator FEV_1_/forced vital capacity (FVC) ≤ 0.70; and no history of asthma, atopy (as assessed by an allergy skin prick test during screening) or any other active lung disease. Patients on home oxygen or with raised carbon dioxide tension (>44 mmHg), *α*_1_-antitrypsin deficiency, recent exacerbation (in the last 4 weeks), an uncontrolled medical condition, or hypersensitivity to inhaled corticosteroids and bronchodilators were not eligible for the study. All nonsmokers and smokers without airflow limitation met the following criteria: age ≥ 42 and ≤75 years, post-BD FEV1% predicted > 80, no diagnostic cancer, diabetes, cardiovascular disease and hypertension, no use of inhaled or oral corticosteroids in the previous 6 months, no atopy, and no respiratory tract infection 1 month prior to the study. Patient characteristics are in Table 1. The experimental procedures were carried out in accordance with the approved guidelines. All experimental protocols were approved by the Research Ethics Boards of The First Affiliated Hospital, Xi'an Jiaotong University (2015-015) and the informed consent was obtained from all subjects.

### 2.2. PBMC Isolation and RNA Extraction

PBMCs were isolated from venous blood by density gradient centrifugation over Ficoll-Paque PLUS reagent (GE Healthcare, Uppsala, Sweden) and suspended in QIAzol Lysis Reagent (Qiagen, Dusseldorf, Germany). Total RNA was extracted using miRNeasy Mini Kit (Qiagen) according to the manufacturer's procedure. RNA integrity was determined by formaldehyde-denaturing gel electrophoresis. We followed the methods of the previous study [10].

### 2.3. lncRNA and mRNA Microarray

Equal amount of RNA sample from each nonsmokers (*N* = 20), smokers (*N* = 17), or COPD patients (*N* = 14) was pooled, respectively, for lncRNA and mRNA profiling assay using Agilent Human lncRNA + mRNA Array v4.0 system by CapitalBiotech Company, Beijing, China. Each array contained a probe set comprising 40,000 human lncRNA transcripts and 34,000 human mRNAs. These lncRNA and mRNA target sequences were merged from multiple databases including Refseq, UCSC, H-InvDB, Human lincRNA catalog, NRED, lncRNAdb, and RNAdb, and 848 were from the Chen Ruisheng Lab (Institute of Biophysics, Chinese Academy of Science). Briefly, first-strand cDNAs were synthesized from 1 *μ*g of total RNA using random or poly T primers carrying the T7 promoter sequence and the CbcScript II reverse transcriptase. Second strand cDNAs were then synthesized using RNaseH and DNA polymerase. The double-stranded cDNA was column-purified and used as a template to amplify cRNA by in vitro transcription reaction. The amplified cRNAs were purified and reverse transcribed into first-strand cDNA using CbcScript II reverse transcriptase. The second cDNA strand was then synthesized using Klenow enzyme, random primers, regular dNTP, and Cy3- or Cy5-labeled dCTP. The labeled cDNAs were hybridized with an array of analysis. Hybridized slides were then washed and scanned with Agilent Microarray Scanner System (G2565CA).

### 2.4. Data Analysis

The lncRNA and mRNA array data were analyzed for data summarization, normalization, and quality control using GeneSpring V11.5 software (Agilent). To select differentially expressed genes, we used threshold values of ≥2-fold change, and a Benjamini–Hochberg-corrected *p* value of 0.05 performed on technically duplicated dots for each lncRNA. The data were Log2 transformed and median centered by genes using the Adjust Data function of CLUSTER 3.0 software. Further analysis was performed by hierarchical clustering with average linkages. Finally, we performed tree visualization using Java TreeView (Stanford University School of Medicine, Stanford, CA, USA).

### 2.5. Quantitative Reverse Transcription PCR Validation

Independent assays were performed using quantitative reverse transcription PCR (qRT-PCR) on all patient samples for individual lncRNA (*NR_026891.1*, *ENST00000502883.1*, *HIT000648516*, *XR_429541.1*, and *ENST00000597550.1*) and mRNAs (*CXCL16*, *HMOX1*, *SLA2*, and *SIGLEC14*) predicted to be regulated by lncRNAs. Total RNA was extracted using miRNeasy Mini Kit (Qiagen) according to the manufacturer's procedure. Quality control and RNA concentrations were determined by spectrophotometer (IMPLEN, Munich, Germany). The reverse transcription was performed on 500 ng of total RNA by using the iScript™ cDNA Synthesis kit (Bio-Rad). The cDNA was then amplified by using the CFX Connect™ Real-Time PCR Detection System (Bio-Rad) with SYBR Green (Bio-Rad) and the primers listed in Table 2. In addition, the expression of the aforementioned lncRNA was detected on the isolated different cell types including CD4^+^ T cells, CD8^+^ T cells, CD14^+^ monocytes, and CD20^+^ B cells from PBMCs in some smokers and COPD patients by positive selection (Anti-PE MicroBeads UltraPure, Miltenyi Biotec, Teterow, Germany). Data were presented relative to *β*-actin for lncRNAs and mRNAs based on calculations of 2^−ΔΔCt^. Statistical significance was defined as *P* < 0.05 as measured by the *t*-test using GraphPad Prism 5 software (GraphPad, San Diego, CA, USA).

### 2.6. lncRNA Target Prediction and Gene Ontology Analysis

The targets of differentially expressed lncRNAs were identified via cis or transregulatory effects. The validated differentially expressed lncRNAs were selected for target prediction. The genes transcribed within a 10 kb upstream or downstream of lncRNAs were considered as cis target genes. lncRNAs and potential cis target genes were paired and visualized using UCSC genome browser. The transtarget genes were chosen by BLAST software according to the impact of lncRNA binding on complete mRNA molecules. The gene ontology (GO) enrichment of dysregulated mRNAs predicted to be regulated by lncRNAs was analyzed by an integrated functional link enrichment of Gene Ontology or gene sets (http://lego.blueowl.cn/).

### 2.7. Stimulation of Cigarette Smoke Extract

The T lymphocyte cell line 6T-CEM (ATCC, Manassas, VA, USA) was stimulated with 5% cigarette smoke extract (CSE) [13] for 24 h, and the mRNA expression of ENST00000502883.1 and CXCL16 was examined by qRT-PCR.

### 2.8. lncRNA ENST00000502883.1 Knockdown by Small Interfering RNA (siRNA) Transfection

6T-CEM cells were cultured in 24-well plates with a 5~7^∗^10^5^/ml density in RPMI1640 medium containing 10% FBS. The cells were then transfected with siRNA targeting lncRNA *ENST00000502883.1* (sequence: 5′-CAAACGUUCAUGUGAAAGATT-3′; 5′-UCUUUCACAUGAACGUUUGTT-3′) and synthesized by GenePharma, Shanghai, China, using the siRNA transfection reagent (Roche, Penzberg, Germany) for 48 h. The scrambled siRNA (GenePharma) was used as a negative control. Six hours before the end of the experiment, 80 nM of PMA, 1 *μ*g/mL of ionomycin and10 *μ*g/mL of Brefeldin A was added to the culture. The efficiency of knockdown was examined by qRT-PCR. The expression of target gene *CXCL16* was detected by qRT-PCR and Western blot.

### 2.9. Western Blot

The protein samples of 6T-CEM cells were loaded (5 *μ*g) on a 10% acrylamide SDS-PAGE gel (Bio-Rad, Hercules, CA, USA) for protein separation, followed by transfer to PVDF membranes (Bio-Rad). The blots were then blocked with 1% BSA in 0.1% Tween 20/PBS for 1 h at room temperature and then incubated overnight at 4°C with antibodies specific for CXCL16 (PeproTech, Rocky Hill, CT). After washing with 0.1% Tween 20 in PBS, the membranes were incubated with a 1 : 3000 dilution of goat anti-rabbit IgG HRP (Calbiochem) in 1% solution of powdered milk in PBS/0.1% Tween 20. The membranes were exposed to ECL solution (Bio-Rad) and imaged by chemiluminescence (Clinx Science Instrument, Shanghai, China).

### 2.10. Chemotaxis Analysis

6T-CEM cells were transfected with lncRNA *ENST00000502883.1* siRNA or scrambled siRNA for 48 h, the supernatant was collected for chemotaxis analysis. The PBMCs were isolated and suspended at a concentration of 10^6^ cells/ml in chemotaxis buffer (RPMI 1640 containing 25 mM Hepes and 1% (*v*/*w*) endotoxin-free bovine serum albumin). The chemotaxis protocol was performed using a 48-well microchemotaxis Boyden chamber (Neuro Probe, Cabin John, MD) with 5 *μ*m pore polycarbonate filters (Neuro Probe). The inferior wells were loaded with cell culture supernatants pretreated at 37°C for 30 min with neutralizing Ab against CXCL16 (R&D systems, Minneapolis, MN, USA) or goat IgG isotype control (R&D systems), chemotaxis buffer, and CXCL12 (PeproTech) at 10^−7^ M were used as negative and positive controls, respectively. 20 ng/ml of CXCL16 (PeproTech) pretreated with neutralizing Ab against CXCL16 (R&D systems) or goat IgG isotype control (R&D systems) was loaded into the inferior wells as well. The chemotaxis system was conducted for 2 h 30 min at 37°C in 5% CO_2_. Each condition was performed in triplicate. Cells having migrated through the filter were counted in the inferior well, and results were expressed as index of chemotaxis compared with chemotaxis buffer.

### 2.11. Statistical Analysis

Statistical analysis for expression of lncRNA and mRNA by qRT-PCR in PBMCs was performed by Mann-Whitney *U* test. The paired *t*-test was performed for cell culture experiments and chemotaxis analysis. Probability values of *P* < 0.05 were considered significant. Data analysis was performed by using the GraphPad prism 5 software (GraphPad, San Diego, CA, USA).

## 3. Results

### 3.1. lncRNA Microarray

**Figure 1:**
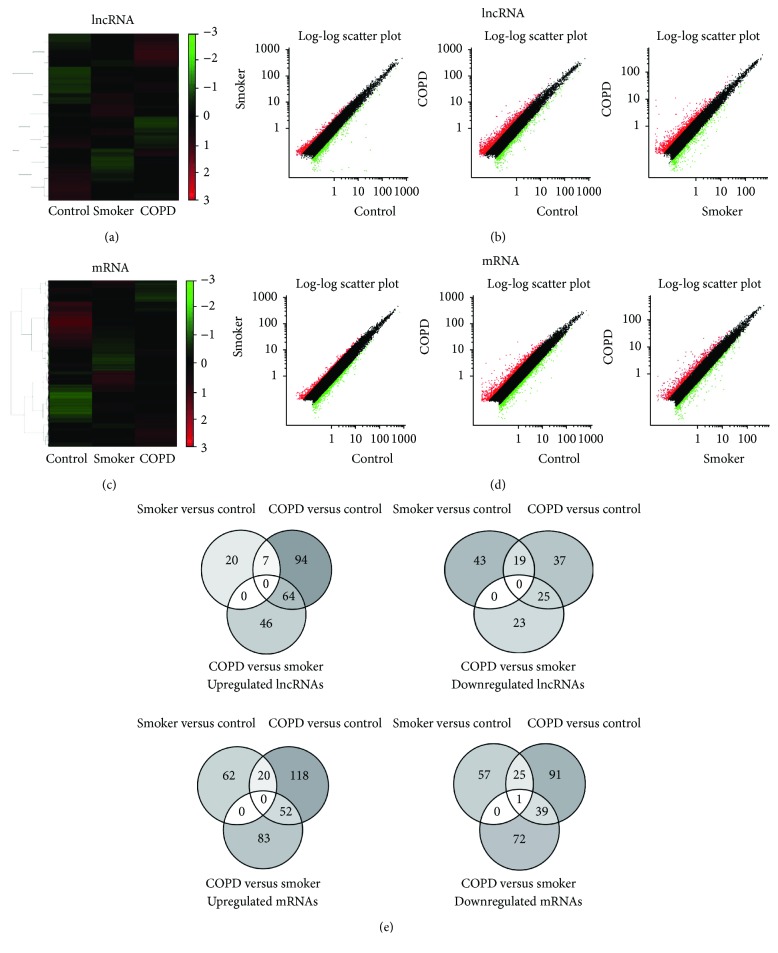
Hierarchical clustering, scatter plot result, and Venn diagram of differentially expressed lncRNAs and mRNAs in PBMCs from nonsmokers, smokers, and COPD patients. (a) Hierarchical clustering image of lncRNA expression of pooled RNA samples from PBMCs. (b) Scatter plot of lncRNA expression of PBMCs. (c) Hierarchical clustering image of mRNA expression of pooled RNA samples from PBMCs. (d) Scatter plot of mRNA expression of PBMCs. Red and green colored dots represent up- and downregulated miRNAs in scatter plot, respectively. (e) Venn diagram of differentially expressed lncRNAs and mRNAs. Figure reproduced from Dang et al. [10], under the Creative Commons Attribution License/public domain.

We firstly examined the lncRNA profiling in PBMCs from 20 nonsmokers, 17 smokers without airflow limitation, and 14 COPD patients. We compared the lncRNA expression between all paired groups (smokers versus nonsmokers, COPD versus nonsmokers, and COPD versus smokers) (Figures 1(a), 1(b), and 1(e)). Compared with nonsmokers, 27 lncRNAs were upregulated and 62 were downregulated in smokers (Table 3), while 165 lncRNAs were upregulated and 81 downregulated in COPD patients (Table 4). When comparing the lncRNA expression between smokers and COPD patients, there were 110 upregulated and 48 downregulated in COPD patients (Table 5).

### 3.2. mRNAs Microarry

We performed the parallel mRNA microarray on pooled RNA samples to compare the mRNA expression profiling in PBMCs from 20 nonsmokers, 17 smokers without airflow limitation, and 14 COPD patients. Eighty-two upregulated and 83 downregulated mRNAs were found in smokers compared with nonsmokers; 190 mRNA were upregulated and 156 downregulated in COPD patients compared with nonsmokers and 135 upregulated and 112 downregulated in COPD patients compared with smokers (Figures 1(c)–1(e), part of the results was published in the previous study [10]).  Table 6 shows the top 10 upregulated and 10 downregulated genes (part of results was published in the previous study [10]).

### 3.3. Target Prediction

**Figure 2:**
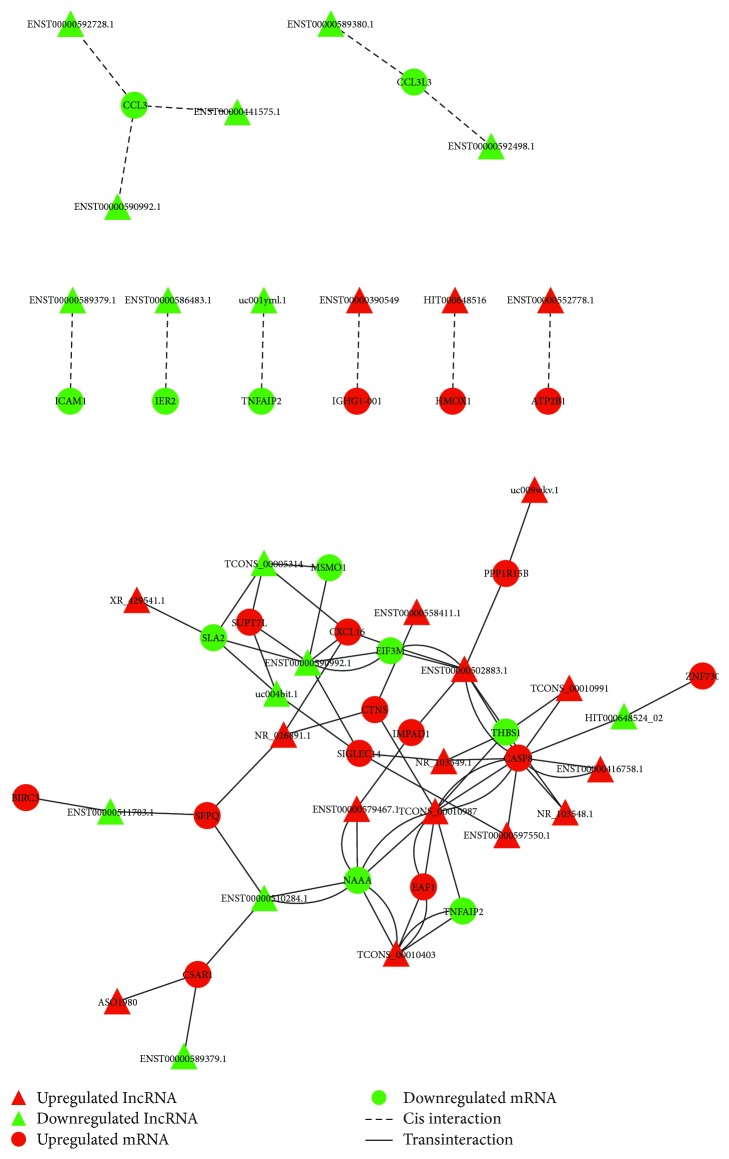
Regulation network between lncRNAs and mRNAs. The regulation of lncRNA on dysregulated mRNAs was predicted and the regulation network was drawn by using Cytoscape software. Red and green color represents up- and downregulated genes, and triangle and circle shape represents cis- and transinteraction, respectively.

We further predicted the potential cis- and transtarget genes for the dysregulated lncRNAs between COPD patients and smokers within the dysregulated mRNAs. The predicted regulation network was shown in Figure 2. The GO enrichment analysis showed that the biological processes and molecular functions including leukocyte migration, immune response, and apoptosis are the main enriched GOs in the dysregulated mRNAs predicted to be regulated by lncRNAs (Table 7). According to the role of target mRNAs in the pathogenesis of COPD, the 5 lncRNAs *NR_026891.1*, *ENST00000502883.1*, *HIT000648516*, *XR_429541.1*, and *ENST00000597550.1* were selected for the further validation (Tables 8 and 9).

### 3.4. qPCR Validation of Dysregulated lncRNAs and mRNAs in PBMCs

**Figure 3:**
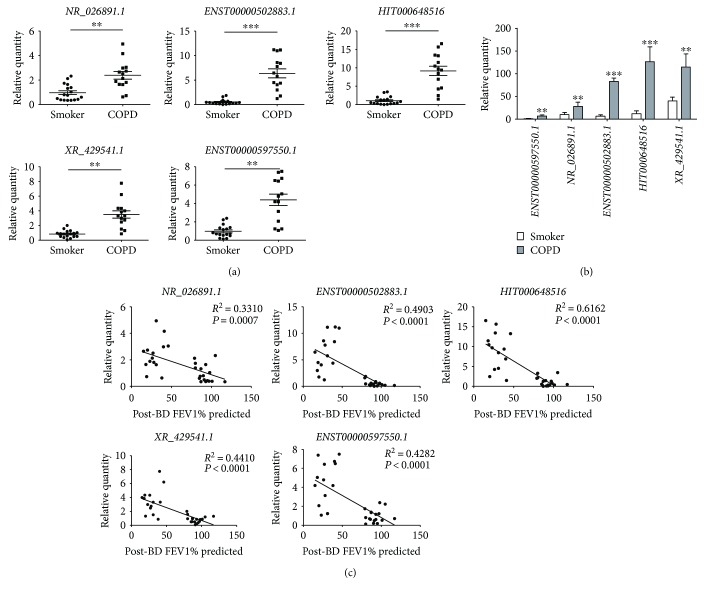
Validation of differentially expressed lncRNAs. (a) qRT-PCR was performed on the same RNA samples (17 smokers and 14 COPD patients) by individual lncRNA for *NR_026891.1*, *ENST00000502883.1*, *HIT000648516*, *XR_429541.1*, and *ENST00000597550.1*. Data are presented as 2^−ΔΔCt^ relative to *β*-actin. ^∗∗^*P* < 0.01 and ^∗∗∗^*P* < 0.001 compared with smokers. (b) Relative abundance of differentially expressed lncRNAs in PBMCs of smokers and COPD patients. ^∗∗^*P* < 0.01 and ^∗∗∗^*P* < 0.001 compared with smokers. (c) Correlation analysis between lncRNA expression and post-BD FEV1% predicted.

The upregulated expression of 5 selected lncRNA (*NR_026891.1*, *ENST00000502883.1*, *HIT000648516*, *XR_429541.1*, and *ENST00000597550.1*) in COPD patients compared with smokers was further validated by qRT-PCR (Figure 3(a)). In PBMCs of smokers, *XR_429541.1* was the most highly expressed lncRNA. In PBMCs of COPD patients, *ENST00000502883.1*, *HIT000648516*, and *XR_429541.1* had the higher expression (Figure 3(b)). To analyze the relationship between lncRNA expression and lung function of subjects, the correlation analysis between lncRNA expression and FEV1% predicted was performed. The significant negative relevance appeared in the expression of these lncRNAs (Figure 3(c)). However, there is no difference in lncRNA sets between ex and current smokers in the smokers and COPD groups (data not shown).

### 3.5. qPCR Validation of Dysregulated lncRNAs in Different Cell Types of PBMCs

**Figure 4:**
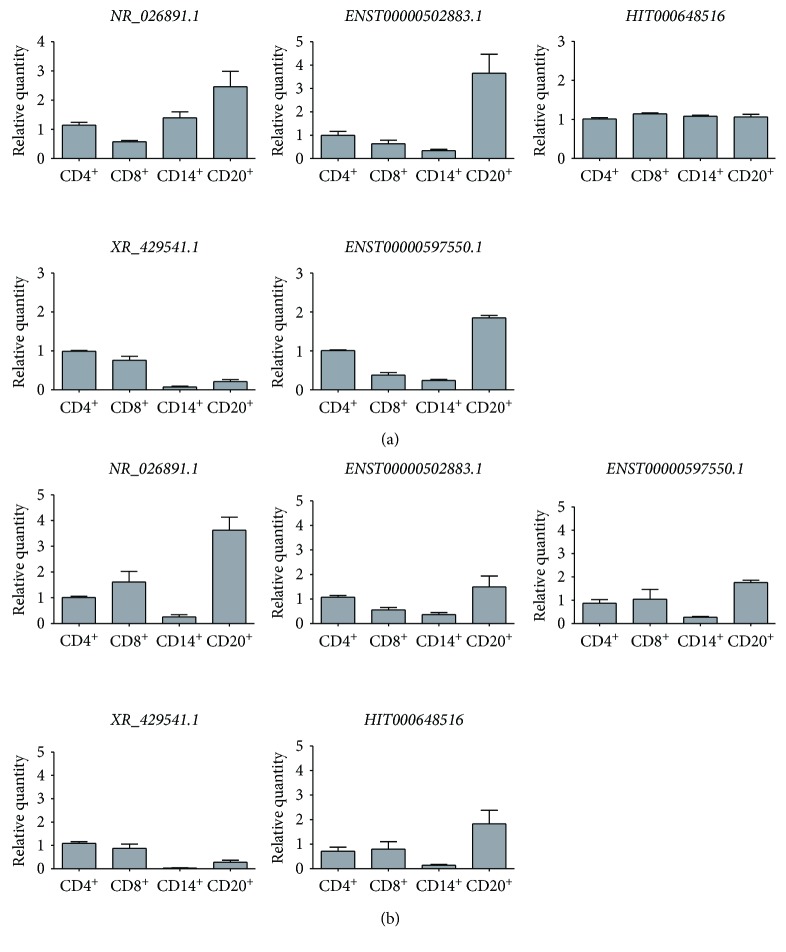
Expression of lncRNAs in the isolated different cell types of PBMCs from smokers (a) and COPD patients (b). The expression of *NR_026891.1*, *ENST00000502883.1*, *HIT000648516*, *XR_429541.1*, and *ENST00000597550.1* was examined by qRT-PCR on CD4^+^ T lymphocytes, CD8^+^ T lymphocytes, CD14^+^ monocytes, and CD20^+^ B lymphocytes from smokers and COPD patients. Data are presented as 2^−ΔΔCt^ relative to *β*-actin.

PBMCs consist mainly of T lymphocytes, B lymphocytes, and monocytes. We therefore analyzed the expression of dysregulated lncRNAs in the isolated different cell types including CD4^+^ T cells, CD8^+^ T cells, CD14^+^ monocytes, and CD20^+^ B cells from PBMCs of smokers (Figure 4(a)) and COPD patients (Figure 4(b)). *NR_026891.1* was highly expressed in monocytes and B cells of smokers, while it was expressed mainly in CD8^+^ T cells and B cells of COPD patients, suggesting CD8^+^ T cells contributed to the increased expression in COPD. *ENST00000502883.1* was consistently expressed higher in B cells and CD4^+^ T cells. In COPD patients, the expression of *HIT000648516* in monocytes was decreased compared with smokers, suggesting the expression in other cells was increased in COPD patients. *XR_429541.1* was mainly expressed in CD4^+^ and CD8^+^ T cells in both smokers and COPD patients. CD8^+^ T cells mainly contributed to the increased expression of *ENST00000597550.1* in COPD patients.

### 3.6. qPCR Validation of Dysregulated Predicted Target mRNAs in PBMCs

**Figure 5:**
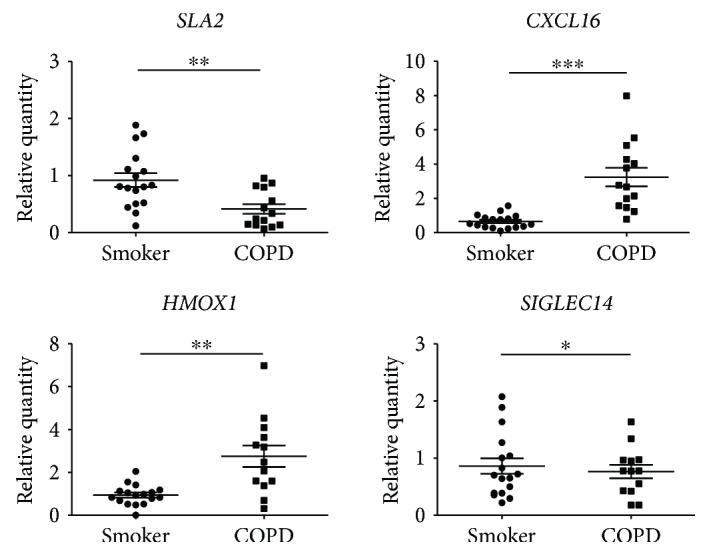
Validation of differentially expressed mRNAs. qRT-PCR was performed on the same RNA samples (17 smokers and 14 COPD patients) by individual mRNA for *CXCL16*, *HMOX1*, *SLA2*, and *SIGLEC14*. Data are presented as 2^−ΔΔCt^ relative to *β*-actin. ^∗^*P* < 0.05, ^∗∗^*P* < 0.01, and ^∗∗∗^*P* < 0.001 compared with smokers.

We further examined the expression of 4 predicted target genes known to be involved in the pathogenesis of COPD in PBMCs from smokers and COPD patients. Consistent with the results of microarray assay, the increased expression of *HMOX1*, *SIGLEC14*, and *CXCL16* and decreased expression of *SLA2* was observed in COPD patients compared with smokers (Figure 5).

### 3.7. Validation of *ENST00000502883.1*-Regulated *CXCL16* Expression in T Cell Line

**Figure 6:**
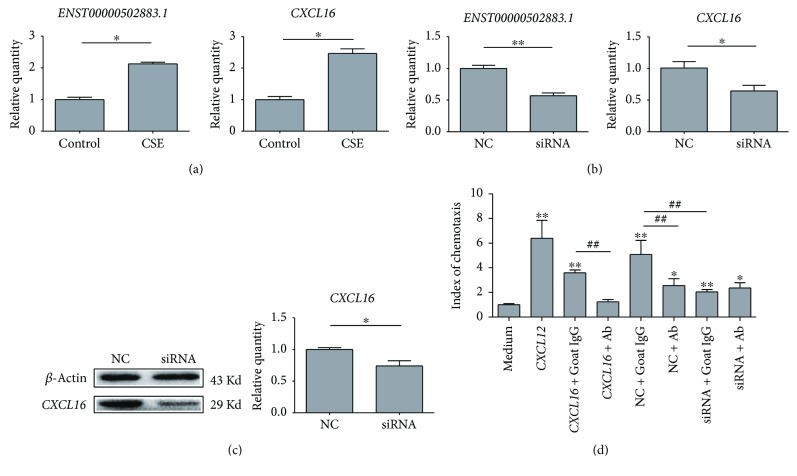
Regulatory role of *ENST00000502883.1* on *CXCL16* expression. (a) Expression of *ENST00000502883.1* on *CXCL16* of 6T-CEM cells under the stimulation of 5% cigarette smoke extract (CSE). Data are presented as 2^−ΔΔCt^ relative to *β*-actin. ^∗^*P* < 0.05. (b) Expression of *CXCL16* on mRNA level of 6T-CEM cells with *ENST00000502883.1* siRNA transfection. Scramble siRNA was used as negative control (NC). Data are presented as 2^−ΔΔCt^ relative to *β*-actin. ^∗^*P* < 0.05. (c) Expression of *CXCL16* on protein level of 6T-CEM cells with *ENST00000502883.1* siRNA transfection. CXCL16 expression was examined by Western blotting. ^∗^*P* < 0.05. (d) The regulatory role of *ENST00000502883.1* on the partially CXCL16-dependent chemotactic effect of 6T-CEM cell supernatant. 6T-CEM cells were transfected with *ENST00000502883.1* siRNA or negative control (NC), and the chemotactic effect of cell culture supernatant on PBMCs was evaluated by a microchemotaxis Boyden chamber. ^∗^*P* < 0.05 and ^∗∗^*P* < 0.01 compared with medium control. ^##^*P* < 0.01 when comparing the two groups with line marks.

We examined the regulatory role of *ENST00000502883.1* on *CXCL16* expression. Firstly, 5% of cigarette smoke extract could significantly enhance the expression of *ENST00000502883.1* and *CXCL16* on T cells (Figure 6(a)). *ENST00000502883.1* knockdown with siRNA consequently decreased the expression of *CXCL16* at both mRNA (Figure 6(b)) and protein (Figure 6(c)) level, suggesting *ENST00000502883.1* could regulate *CXCL16* expression. We then analyzed the chemotactic effect of T cell supernatant on PBMCs. The pretreatment with CXCL16 neutralizing antibody significantly decreased the chemotactic effect of NC control culture medium, suggesting the CXCL16 secreted by T cells play a good biological chemotactic function. Meanwhile, the supernatant of T cells with siRNA transfection had the reduced effect compared with NC control, which indicted lncRNA *ENST00000502883.1* could regulate *CXCL16* expression and furtherl regulate the recruitment of inflammatory cells.

## 4. Discussion

Increasing evidence has confirmed lncRNAs to be one of the most important factors controlling gene expression [14–16]. Therefore, for the first time, we evaluated the lncRNA and mRNA expression profile of PBMCs from healthy nonsmokers, smokers without airflow limitation, and COPD patients. We identified 158 differentially expressed lncRNAs in PBMCs from COPD subjects compared with smokers without airflow limitation. The dysregulated expression of 5 selected lncRNA including *NR_026891.1* (*FLJ10038*), *ENST00000502883.1* (*RP11-499E18.1*), *HIT000648516*, *XR_429541.1*, and *ENST00000597550.1* (*CTD-2245F17.3*) were validated by qPCR. The GO enrichment analysis for the dysregulated mRNAs predicted to be regulated by lncRNAs showed that leukocyte migration, immune response, and apoptosis are the main enriched processes that previously reported to be involved in the pathogenesis of COPD. We further validated the dysregulation of target genes *CXCL16*, *HMOX1*, *SLA2*, and *SIGLEC14* in PBMCs of COPD patients. It is noted that the predicted target genes discussed in the present study did not overlay the ones that we predicted in the study of dysregulated miRNAs of COPD patients [10]. This may suggest that the regulation profile of lncRNA and miRNA on target genes is different in PBMCs of COPD patients.

For the economic consideration, the equal amount of RNA sample from each smoker and COPD patients was pooled, respectively, for lncRNA profiling assay. And the expression of selected lncRNAs in each individual was further validated by qRT-PCR. The similar approach was previously used on lncRNA microarray assays [17–19].

CXCL16 was reported as one of the systemic inflammatory markers for COPD in a large cohort of COPD patients and controls [20]. In this study, increased CXCL16 expression was also found in PBMCs of COPD patients versus smokers. CXCL16 is expressed by dendritic cells, macrophages, T cells, and B cells and can act as a chemoattractant for Th1 cells, which implies its relevance to COPD [21, 22]. *CXCL16* was predicted to be regulated by *NR_026891.1* (*FLJ10038*) and *ENST00000502883.1* (*RP11-499E18.1*) through a transregulation manner. Few studies have been reported on the two lncRNAs except for the upregulation of *NR_026891.1* (*FLJ10038*) in an experimental model of Alzheimer's disease [23]. In this study, we confirmed the regulatory role of *ENST00000502883.1* on *CXCL16* expression and consequently the effect on PBMC recruitment.

The lncRNA *HIT000648516* was predicted to target the *HMOX1* gene by a sense manner. *HMOX1* gene encodes heme oxygenase (HO)-1, which is induced under physiological conditions such as inflammation and oxidative stress and protects against inflammatory- and oxidant-mediated cellular injury [24]. HO-1 may also play a vital function in maintaining cellular homeostasis [25]. Moreover, the increased expression of HO-1 was found in sputum samples obtained at the onset of a severe COPD exacerbation [26] and in the peripheral blood monocytes during acute inflammatory illnesses of children [27]. Thus, the increased expression of HO-1 in PBMCs may reflect a protective response in COPD patients under the environment of inflammation and oxidative stress.

The expression of *SLA2* gene was decreased in PBMCs of COPD patients compared with smokers, and lncRNA *XR_429541.1* was predicted to regulate this gene by transregulation. The *SLA2* gene encodes Src-like adaptor protein-2 (SLAP-2), which shares 36% sequence similarity with *SLAP*. SLAP-2 is predominantly expressed in hematopoietic cells and plays an inhibitory role in the activation of T cells [28]. In COPD, once activated, T cells are present in the lung and exert their effector functions by attracting other inflammatory cells like neutrophils and macrophages and enhancing their inflammatory functions [8]. Therefore, the downregulation of *SLAP-2* in PBMCs seen in this study may be related with inflammation in COPD patients.

The *SIGLEC14* gene expression was predicted to be regulated by *ENST00000597550.1* (*CTD-2245F17.3*). Siglecs are a family of sialic acid-binding lectins expressed mainly on innate immune cells [29]. Siglec-14, a Siglec family member with an activating signaling property, is expressed on granulocytes and monocytes. Siglec-14 serum concentration can serve as a useful marker for COPD exacerbation susceptibility and consequential decline in pulmonary function [30]. Patients with COPD who are homozygous null for this allele had fewer inflammatory exacerbations than patients expressing the wild-type allele, which suggests that Siglec-14 may promote inflammatory sequelae caused by neutrophils [30]. Furthermore, inhaled corticosteroids may exert negative effects on treatment through increased Siglec-14 expression [31].

Overall, through lncRNA and mRNA expression profiling in nonsmokers, smokers, and COPD patients, we identified the dysregulated lncRNAs and mRNAs in PBMCs from COPD patients compared to smokers. We further analyzed the regulation network between lncRNAs and mRNAs, where the genes *CXCL16*, *HMOX1*, *SLA2*, and *SIGLEC14* were predicted to be regulated by certain lncRNAs through sense or miRNA regulation. This study may provide clues for further studies targeting lncRNAs to control inflammation in COPD.

## Figures and Tables

**Table 1:** Clinical characteristics of nonsmokers, smokers without airflow limitation, and COPD patients.

	Healthy nonsmokers	Smokers without COPD	COPD
Number	20	17	14
Age	61 ± 14	56 ± 17	69 ± 8
Male/female	12/8	17/0	13/1
Current/ex-smokers	—	14/3	8/6
Post-BD FEV1% predicted	101.0 ± 8.6	92.9 ± 9.0	29.0 ± 10.6
Gold stage			
I		—	0
II		—	6
III-IV		—	8

Data are presented as mean ± SD. BD: bronchodilator; FEV1, forced expiratory volume in 1 s.

**Table 2:** The sequence of primers for real-time PCR.

Gene	Sequence (5′-3′)	Direction
*NR_026891.1*	GGACCACATCTCCTGTACCA	Forward
	CCTCATGACGTGCACTTTACC	Reverse
*ENST00000502883.1*	GTGTCCATGTAACTAACTCCTGGT	Forward
	GTCAGCGGGAAGGAAGACAG	Reverse
*HIT000648516*	ATAGAAGCGATCTACCCTCACAG	Forward
	TGCTGGGCTCGTTCGT	Reverse
*XR_429541.1*	TGGTCACTTTCCAGTTTCCACA	Forward
	AATTCAGATCCCACATCAGCCT	Reverse
*ENST00000597550.1*	GCCCTCCACCCCAGATTAAC	Forward
	CTCTTTCTTCTCTTCTGGACTTCT	Reverse
*CXCL16*	CTGAGAGCTTACCATCGGTGT	Forward
	TCAAGACAGCTCATCAATTCCT	Reverse
*HMOX1*	GGCCAGCAACAAAGTGCAAG	Forward
	ATGGCATAAAGCCCTACAGCA	Reverse
*SLA2*	GACATCTGCTGCCTACTCAAGG	Forward
	TGTGGCAGCTTCAGAAAACAGG	Reverse
*SIGLEC14*	CTGCACAGTTGACAGCAACC	Forward
	GTCTGGGAAGGATTGAGGGC	Reverse
*β-Actin*	TACCTCATGAAGATCCTCACC	Forward
	TTTCGTGGATGCCACAGGAC	Reverse

**Table 3:** Top 10 upregulated and downregulated lncRNAs in smokers compared with nonsmokers.

lncRNA	Fold change	*P* value
Upregulation		
*ENST00000594469.1*	4.132	0.0049
*TCONS_00017656*	2.708	0.0073
*ENST00000452347.1*	2.610	0.0076
*ENST00000445076.1*	2.523	0.0080
*ENST00000517658.1*	2.495	0.0081
*ENST00000379928.4*	2.488	0.0082
*ENST00000425031.1*	2.469	0.0083
*ENST00000571404.1*	2.398	0.0086
*ENST00000417071.1*	2.392	0.0087
*ENST00000447643.1*	2.372	0.0088
Downregulation		
*ENST00000602863.1*	0.004	0.0028
*ENST00000445814.1*	0.018	0.0029
*TCONS_00017343*	0.047	0.0031
*ENST00000602587.1*	0.059	0.0031
*ENST00000553269.1*	0.154	0.0040
*uc.173*	0.181	0.0044
*ENST00000420213.1*	0.207	0.0047
*TCONS_00002106*	0.220	0.0049
*ENST00000502883.1*	0.221	0.0049
*ENST00000434051.1*	0.226	0.0050

**Table 4:** Top 10 upregulated and downregulated lncRNAs in COPD patients compared with nonsmokers.

lncRNA	Fold change	*P* value
Upregulation		
*ENST00000594469.1*	20.223	0.0049
*ENST00000416105.1*	19.604	0.0030
*XR_428545.1*	18.063	0.0030
*NR_103548.1*	8.289	0.0034
*TCONS_00010984*	7.562	0.0035
*TCONS_00010403*	7.522	0.0035
*ENST00000416758.1*	7.113	0.0035
*HIT000064697*	5.478	0.0038
*ENST00000513492.1*	5.292	0.0039
*ENST00000607854.1*	4.622	0.0041
Downregulation		
*TCONS_00009962*	0.133	0.0041
*ENST00000456917.1*	0.134	0.0041
*ENST00000609385.1*	0.136	0.0041
*ENST00000517983.1*	0.156	0.0045
*ENST00000420213.1*	0.199	0.0053
*TCONS_00005314*	0.209	0.0055
*ENST00000584923.1*	0.212	0.0055
*TCONS_00008360*	0.221	0.0057
*ENST00000445814.1*	0.238	0.0062
*ENST00000602813.1*	0.239	0.0062

**Table 5:** Top 10 upregulated and downregulated lncRNAs in COPD patients compared with smokers.

lncRNA	Fold change	*P* value
Upregulation		
*ENST00000602863.1*	79.144	0.0028
*ENST00000446595.1*	19.715	0.0030
*TCONS_00016340*	17.902	0.0030
*XR_428545.1*	14.426	0.0031
*TCONS_00017343*	13.416	0.0031
*ENST00000416105.1*	13.057	0.0032
*ENST00000445814.1*	12.971	0.0032
*TCONS_00009234*	7.267	0.0036
*ENST00000434051.1*	6.920	0.0036
*ENST00000416758.1*	6.476	0.0037
Downregulation		
*TCONS_00028904*	0.056	0.0032
*ENST00000517983.1*	0.092	0.0035
*ENST00000456917.1*	0.105	0.0037
*ENST00000609385.1*	0.115	0.0038
*TCONS_00005314*	0.187	0.0048
*HIT000648524_02*	0.211	0.0053
*TCONS_00009962*	0.216	0.0053
*TCONS_00008360*	0.223	0.0055
*XR_429946.1*	0.264	0.0065
*ENST00000548760.2*	0.268	0.0066

**Table 6:** Top 10 upregulated and downregulated mRNAs in COPD patients compared with smokers.

Gene symbol	Gene name	Fold change Smokers versus nonsmokers (*P* value)	Fold change COPD versus nonsmokers (*P* value)	Fold change COPD versus smokers (*P* value)	Function
Upregulation					
*CD177*	CD177 molecule	3.48 (0.0056)	78.70 (0.0029)	22.59 (0.0030)	Leukocyte migration
*MUC17*	Mucin 17,cell surface associated	1.08 (0.2270)	23.22 (0.0030)	21.54 (0.0030)	Extracellular matrix constituent
*IL1R2*	Interleukin 1 receptor, type II	0.92 (0.7332)	10.08 (0.0033)	10.96 (0.0033)	Decoy receptor, inhibits the activity of IL-1
*SARDH*	Sarcosine dehydrogenase	0.19 (0.0045)	1.83 (0.0100)	9.52 (0.0033)	Mitochondrial matrix
*EGR3*	Early growth response 3	1.12 (0.1549)	7.57 (0.0035)	6.75 (0.0036)	Positive regulation of endothelial cell proliferation
*AREG*	Amphiregulin	0.65 (0.0319)	4.01 (0.0071)	6.16 (0.0048)	EGF family, promote the growth of normal epithelial cells
*SLC6A2*	Solute carrier family 6 (neurotransmitter transporter, noradrenalin), member 2	0.39 (0.0093)	1.84 (0.0099)	4.70 (0.0042)	Sodium symporter
*TMEM167A*	Transmembrane protein 167A	0.79 (0.8343)	3.68 (0.0435)	4.55 (0.0077)	Golgi apparatus
*KCNJ15*	Potassium inwardly-rectifying channel, subfamily J, member 15	0.78 (0.5713)	3.45 (0.0049)	4.42 (0.0043)	Potassium channel activity
*FCHO1*	FCH domain only 1	0.26 (0.0057)	1.15 (0.0381)	4.39 (0.0043)	Clathrin-mediated endocytosis
Downregulation					
*IL1A*	Interleukin 1, alpha	2.03 (0.0117)	0.05 (0.0031)	0.02 (0.0029)	Immune response
*IL6*	Interleukin 6 (interferon, beta 2)	1.59 (0.0111)	0.10 (0.0044)	0.06 (0.0032)	Proinflammatory and anti-inflammatory role
*CXCL10*	Chemokine (C-X-C motif) ligand 10	1.06 (0.2735)	0.07 (0.0033)	0.07 (0.0033)	Leukocyte chemotaxis
*TNF*	Tumor necrosis factor	0.43 (0.0112)	0.04 (0.0030)	0.085 (0.0035)	Inflammation, cause apoptosis
*CCL20*	Chemokine (C-C motif) ligand 20	1.06 (0.2724)	0.13 (0.0041)	0.13 (0.0039)	Lymphocytes chemotaxis
*CCL4*	Chemokine (C-C motif) ligand 4	0.87 (0.3686)	0.14 (0.0045)	0.16 (0.0047)	Leukocyte chemotaxis
*CCL3L3*	Chemokine (C-C motif) ligand 3-like 3	0.89 (0.5755)	0.17 (0.0066)	0.19 (0.0063)	Leukocyte chemotaxis
*C9orf7*	Chromosome 9 open reading frame 7	1.40 (0.0126)	0.27 (0.0070)	0.19 (0.0049)	Calcium channel activity
*IL1RN*	Interleukin 1 receptor antagonist	0.90 (0.5833)	0.19 (0.0051)	0.21 (0.0053)	Inhibition of the activities of IL-1
*RNF19B*	Ring finger protein 19B	2.21 (0.0099)	0.53 (0.0362)	0.24 (0.0059)	Cytotoxic effects of natural killer (NK) cells

Table reproduced from Dang et al. [10], under the Creative Commons Attribution License/public domain.

**Table 7:** Top 10 enriched GOs of targeted genes predicted to be regulated by dysregulated lncRNAs in COPD patients compared to smokers.

GO	*P* value	Enrich score	Gene list
GO:0034612 response to tumor necrosis factor	0.000275	26.766	*CCL3*, *THBS1*, *CXCL16*, and *CASP8*
GO:0002685 regulation of leukocyte migration	0.00241	22.613	*HMOX1*, *CCL3*, and *THBS1*
GO:1,902,041 regulation of extrinsic apoptotic signaling pathway via death domain receptors	0.00252	41.244	*THBS1* and *HMOX1*
GO:2,001,236 regulation of extrinsic apoptotic signaling pathway	0.00494	15.411	*HMOX1*, *THBS1*, and *CASP8*
GO:0002443 leukocyte-mediated immunity	0.00494	14.489	*SLA2*, *IGHG1–001*, and *CCL3*
GO:0002757 immune response-activating signal transduction	0.00494	9.249	*SLA2*, *C5AR1*, *IGHG1–001*, *CASP8*, and *BIRC3*
GO:0002687 positive regulation of leukocyte migration	0.00494	23.266	*CCL3* and *THBS1*
GO:2,001,233 regulation of apoptotic signaling pathway	0.00494	8.922	*HMOX1*, *SFPQ*, *THBS1*, and *CASP8*
GO:0071356 cellular response to tumor necrosis factor	0.00494	20.39	*CCL3* and *THBS1*
GO:0010035 response to inorganic substance	0.00494	8.309	*HMOX1*, *PPP1R15B*, *THBS1*, and *CASP8*

**Table 8:** Selected dysregulated lncRNAs in COPD patients compared with smokers.

lncRNA	Fold change Smokers versus nonsmokers	Fold change COPD versus nonsmokers	Fold change COPD versus smokers
*NR_026891.1*	1.15	2.76	2.39
*ENST00000502883.1*	0.22	0.78	3.51
*HIT000648516*	0.51	1.14	2.25
*XR_429541.1*	1.13	2.78	2.46
*ENST00000597550.1*	0.79	1.61	2.05

**Table 9:** The selected predicted regulation of dysregulated lncRNAs on mRNAs in COPD patients compared to smokers.

lncRNA	Gene symbol	Target gene	Microarray analyze fold change	Function
*NR_026891.1*	*FLJ10038*	*CXCL16*	2.29	Induced by the inflammatory cytokines IFN-gamma and TNF-alpha; cytokine-cytokine receptor interaction, organism-specific biosystem
*ENST00000502883.1*	*RP11-499E18.1*	*CXCL16*	2.29	Induced by the inflammatory cytokines IFN-gamma and TNF-alpha; cytokine-cytokine receptor interaction, organism-specific biosystem
*HIT000648516*	*—*	*HMOX1*	2.71	Keap1-Nrf2 pathway, organism-specific biosystem
*XR_429541.1*	*—*	*SLA2*	0.46	Negative regulation of B cell activation; regulation of immune response TCR signaling in naive CD4^+^ T cells
*ENST00000597550.1*	*CTD-2245F17.3*	*SIGLEC14*	2.57	Innate immune system Cell adhesion
